# Supplementary figures and images for: Mitochondrial genome characteristics of six Phylloscopus species and their phylogenetic implication
Source: PeerJ. 2023 Oct 11;11:e16233. doi: 10.7717/peerj.16233 (PMC10576491; doi:10.7717/peerj.16233)

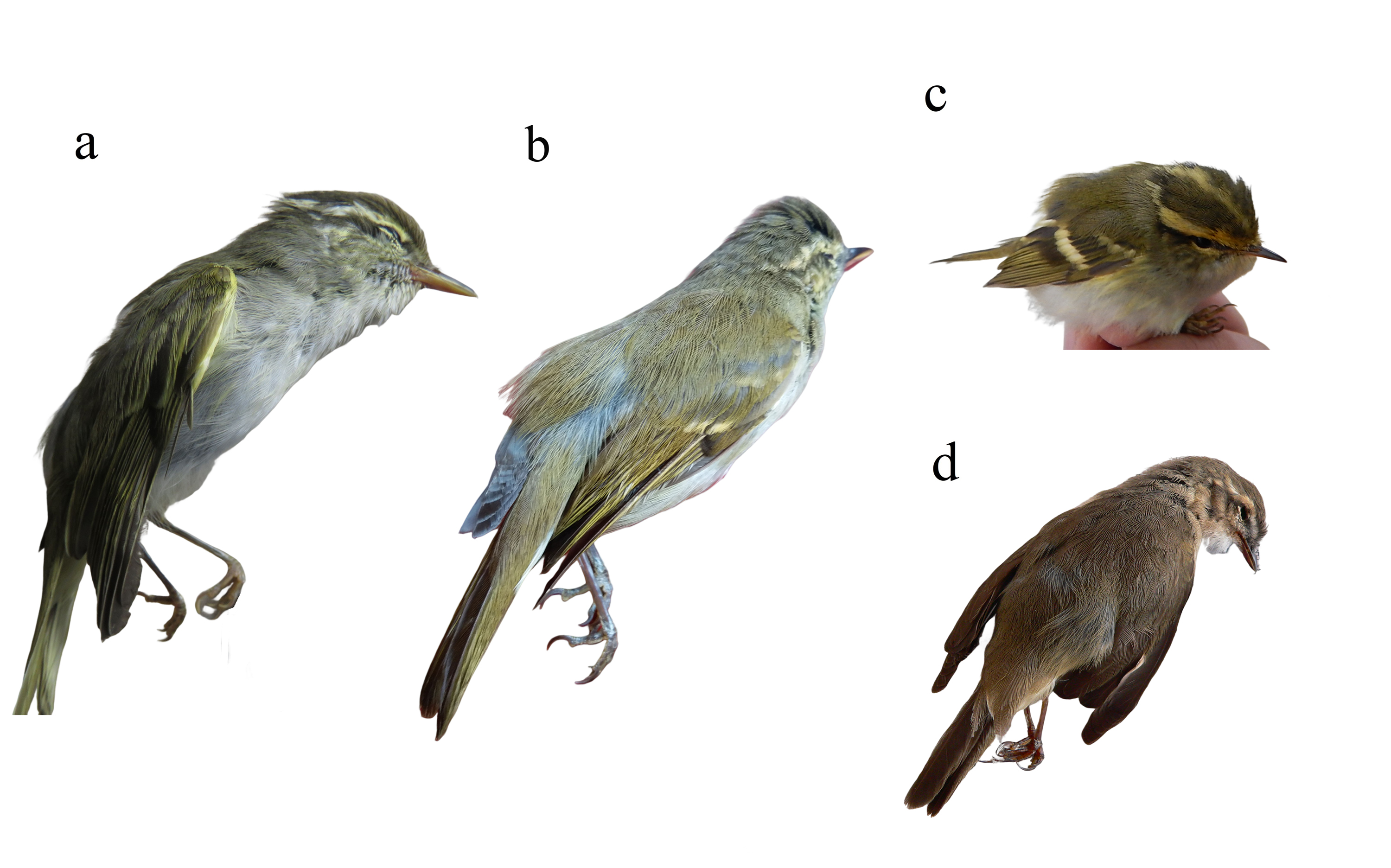

Supplement: Supplemental Information 1 — Note: (A): P. borealis, (B): P. trochiloides, (C): P. proregulus, (D): P. fuscatus. Photos of P. burki and P. reguloides were not obtained. [file peerj-11-16233-s001.png]

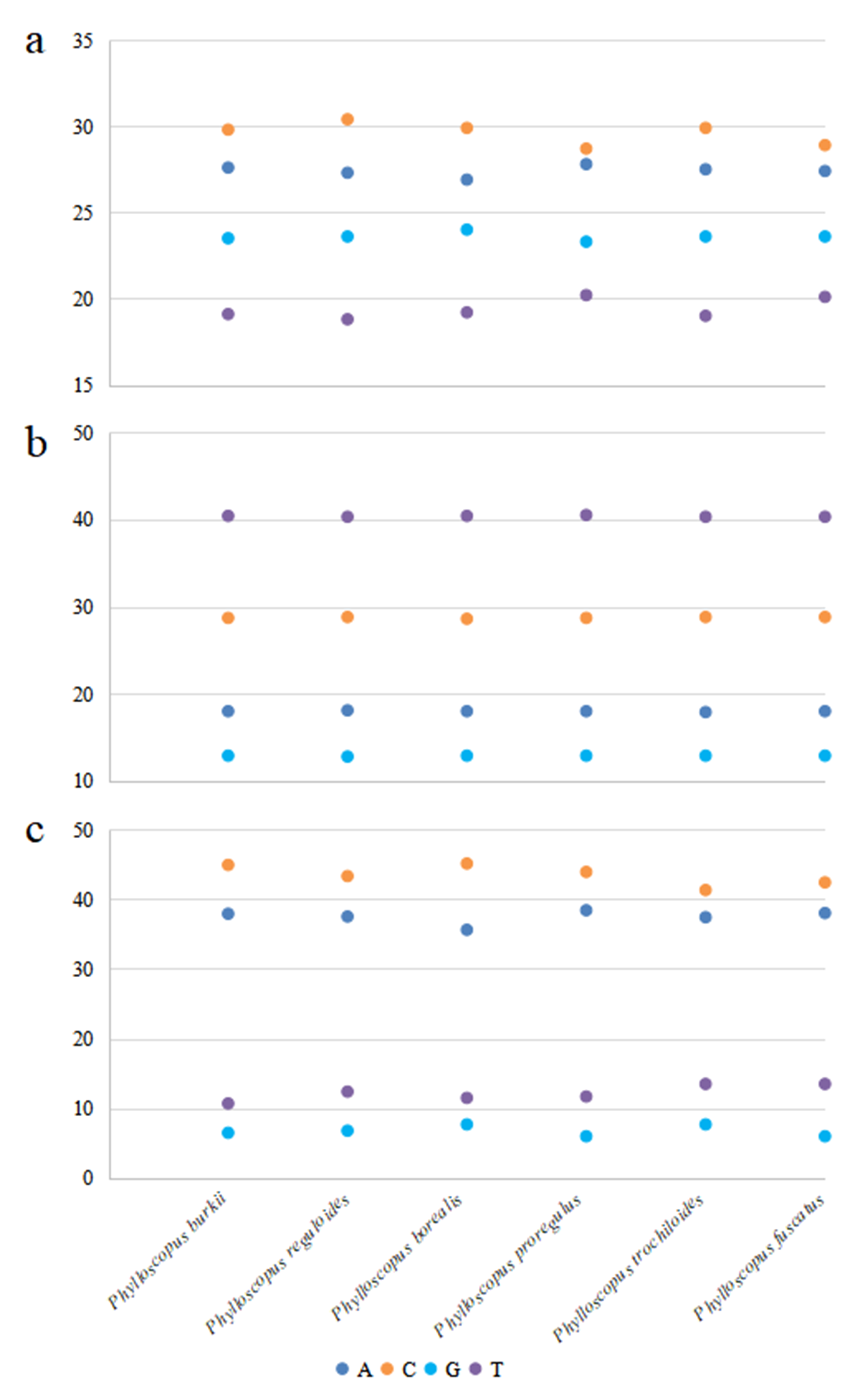

Supplement: Supplemental Information 2 — Note: (A): the first codons, (B): the second codons, (C): the third codons. [file peerj-11-16233-s002.png]

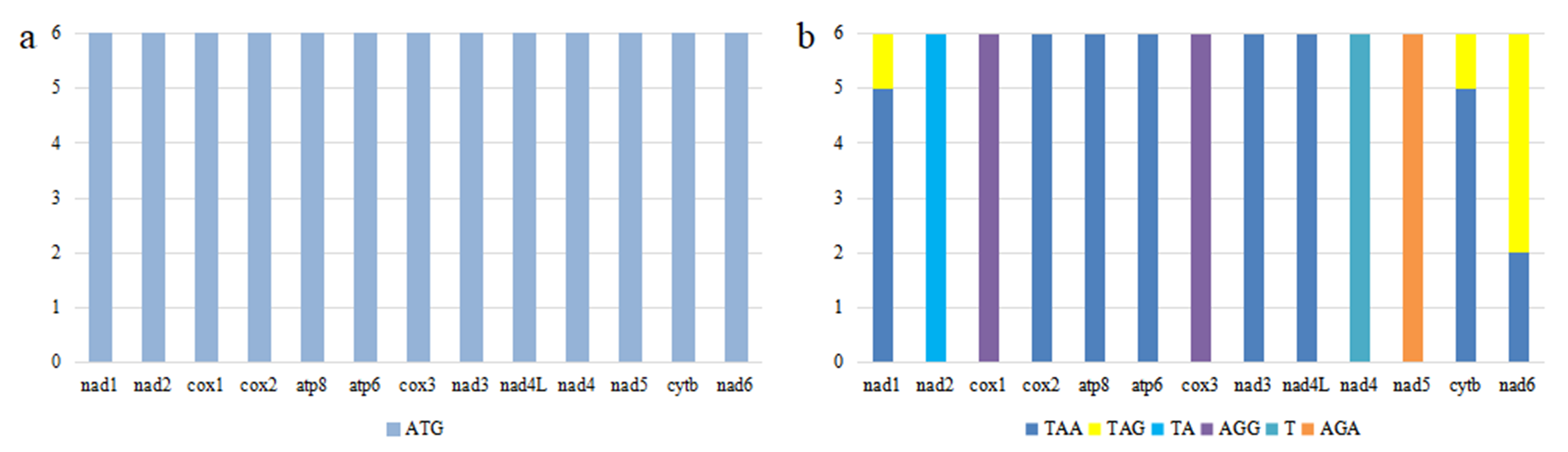

Supplement: Supplemental Information 3 — Note: (A): initiation codon, (B): termination codon. [file peerj-11-16233-s003.png]

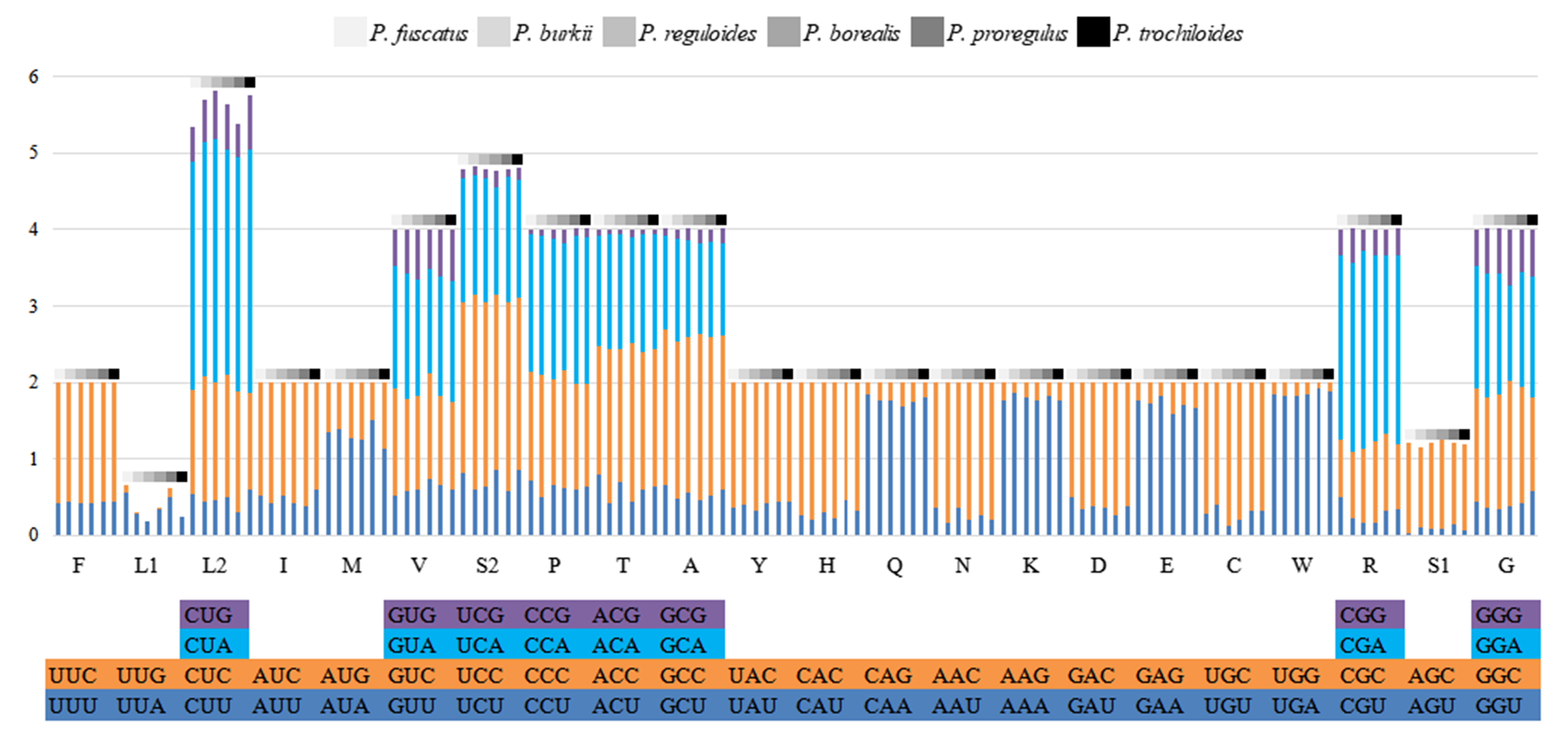

Supplement: Supplemental Information 4 [file peerj-11-16233-s004.png]

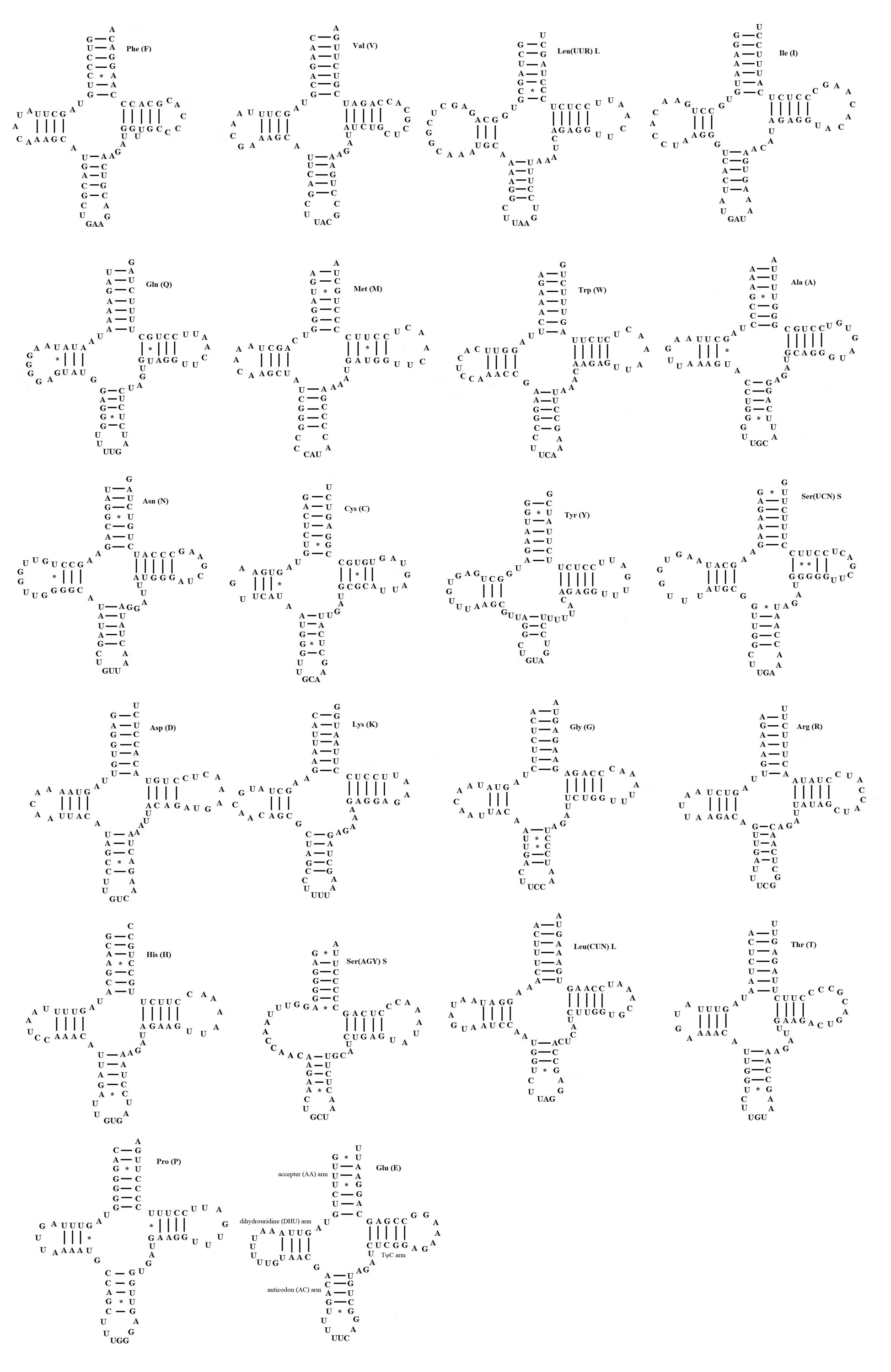

Supplement: Supplemental Information 5 [file peerj-11-16233-s005.png]

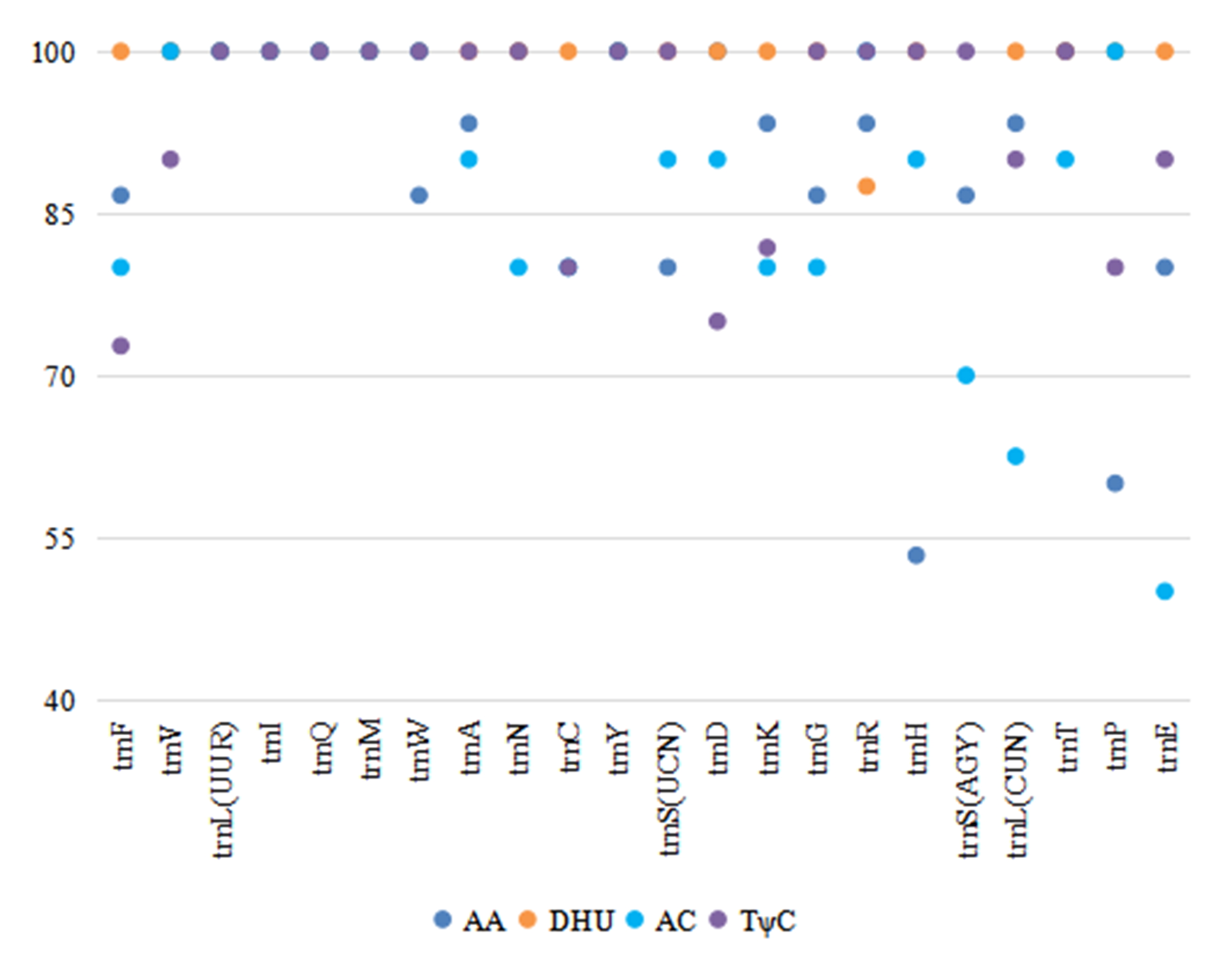

Supplement: Supplemental Information 6 [file peerj-11-16233-s006.png]

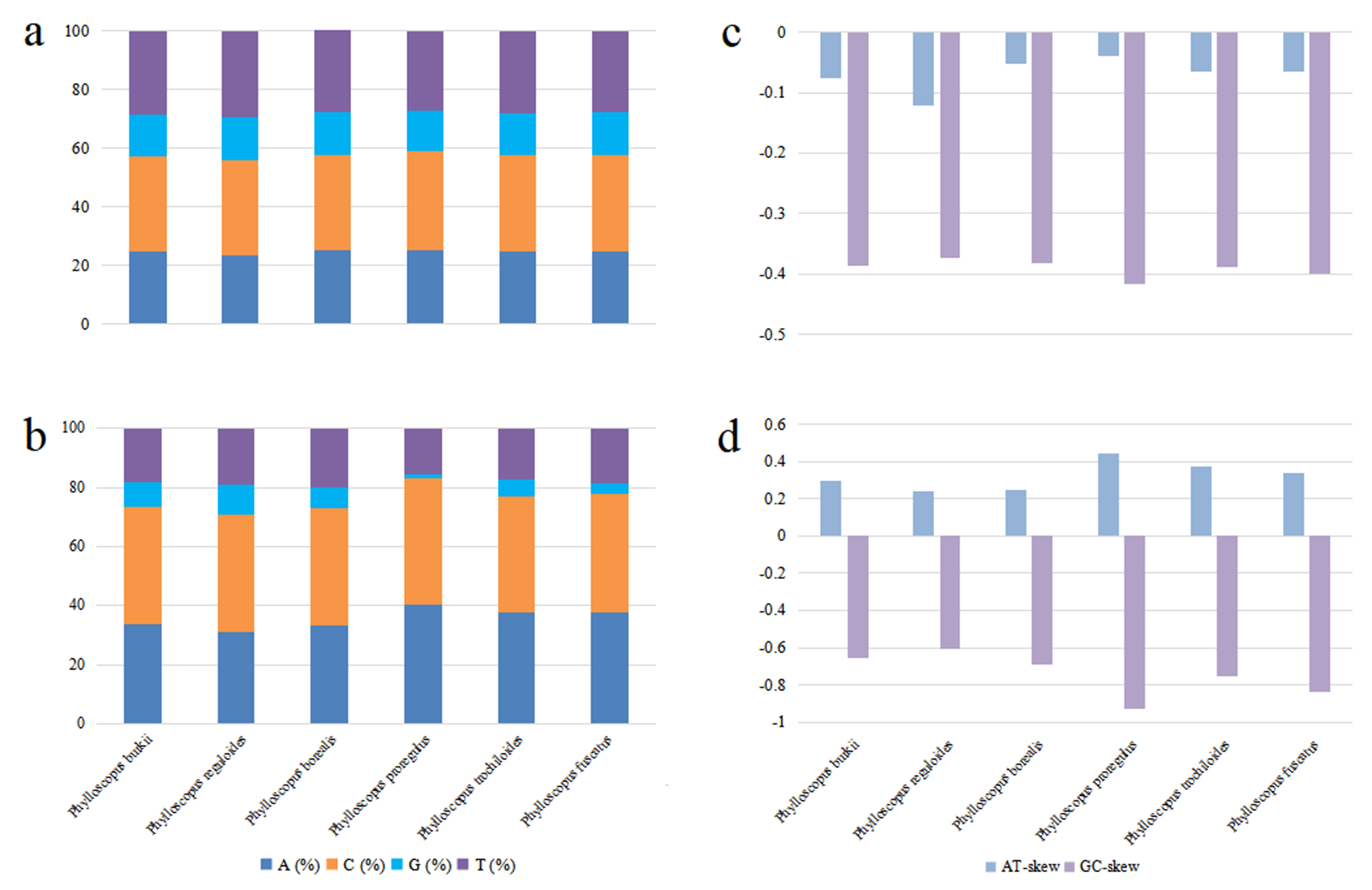

Supplement: Supplemental Information 7 — Note: (A), (C): CR1, (B), (D): remnant CR2. [file peerj-11-16233-s007.png]

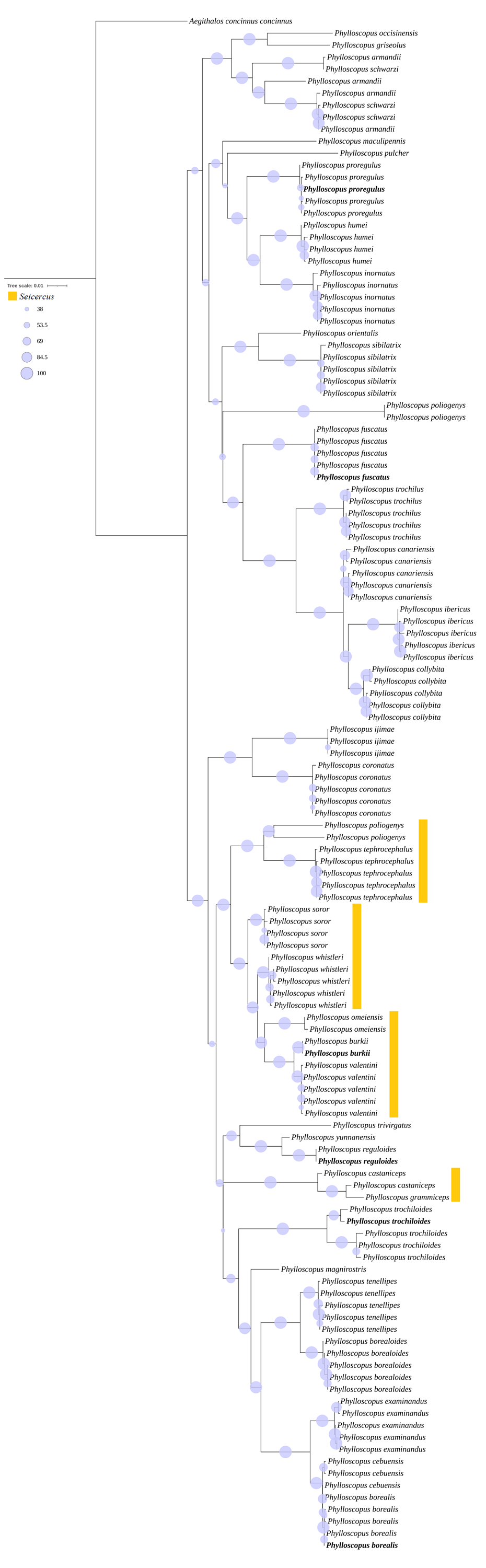

Supplement: Supplemental Information 8 — Note: the newly sequenced mitochondrial sequences labelled with bold format. [file peerj-11-16233-s008.png]

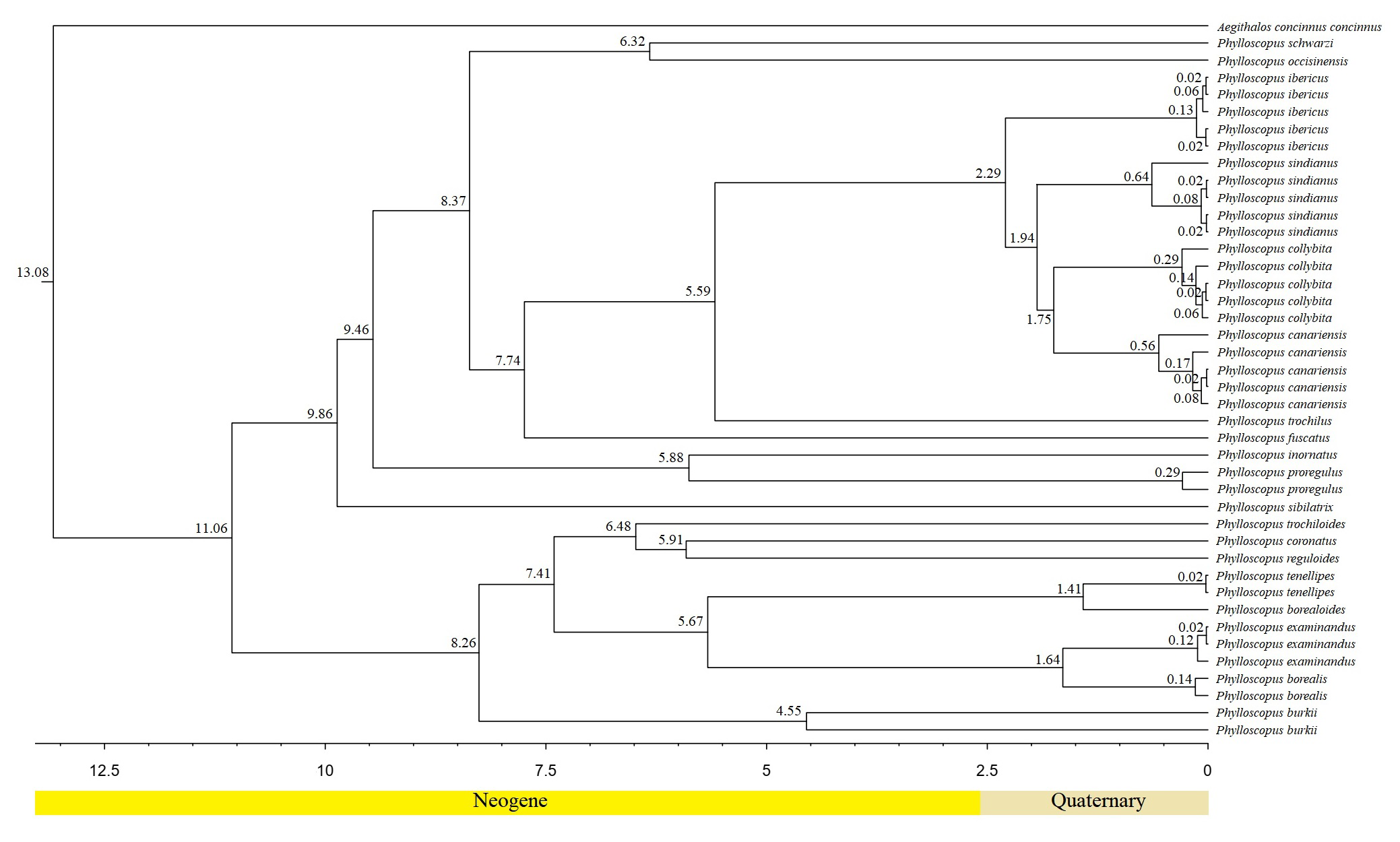

Supplement: Supplemental Information 9 [file peerj-11-16233-s009.png]
