# Supplementary material for: Mitochondrial genome characteristics of six Phylloscopus species and their phylogenetic implication
Source: PeerJ. 2023 Oct 11;11:e16233. doi: 10.7717/peerj.16233 (PMC10576491; doi:10.7717/peerj.16233)
Supplement: Supplemental Information 10 [file peerj-11-16233-s010.docx]

**Table S1** Six sampled *Phylloscopus* species.

| Species | Sampling information |
| --- | --- |
| *Phylloscopus fuscatus* | Hongjiannao, Shaanxi Province, China in 2013 |
| *Phylloscopus burkii* | Wenxian County, Gansu Province, China in 2003 |
| *Phylloscopus reguloides* | Yanbian County, Sichuan Province, China in 2005  Yanbian County |
| *Phylloscopus borealis* | Lantian County, Shaanxi Province, China in 2018 |
| *Phylloscopus proregulus* | Zhouzhi County, Shaanxi Province, China in 2018 |
| *Phylloscopus trochiloides* | Lantian County, Shaanxi Province, China in 2018  Province, China |
