# Supplementary material for: Mitochondrial genome characteristics of six Phylloscopus species and their phylogenetic implication
Source: PeerJ. 2023 Oct 11;11:e16233. doi: 10.7717/peerj.16233 (PMC10576491; doi:10.7717/peerj.16233)
Supplement: Supplemental Information 11 — Note: (A): mitogenome sequences, (B): cox1 gene. [file peerj-11-16233-s011.docx]

**Table S2** The sampled *Phylloscopus* species used for phylogenetic tree reconstruction. Note: a: mitogenome sequences, b: *cox1* gene.

| Species^a^ | Length (bp) | GenBank accession number | Species^b^ | GenBank accession number |
| --- | --- | --- | --- | --- |
| *P. borealis* | 16,898 | OR030350 | *P. armandii* | JQ175806  JQ175805  JQ175804  HQ608871 |
| *P. borealis* | 16,881 | NC_045526 | *P. borealis* | OR030350  GU571553  GU571552  KC354945  JN801360 |
| *P. borealoides* | 16,904 | MN125373 | *P. borealoides* | GQ482409  GQ482415  KY627730  LC087206  AB843690 |
| *P. burkii* | 16986 | OR030349 | *P. burkii* | OR030349  HQ608892 |
| *P. burkii* | 16,963 | KX977449 | *P. canariensis* | MW845058  MW845057  MW845056  MW845010  MW845011 |
| *P. canariensis* | 16,936 | NC_071941 | *P. castaniceps* | HQ608893  KU343222 |
| *P. canariensis* | 16,936 | OP380515 | *P. cebuensis* | JQ175809  JQ175807  EU541460 |
| *P. canariensis* | 16,936 | OP380514 | *P. collybita* | GU572029  GU571555  HQ608872  MF580198  KT803661 |
| *P. canariensis* | 16,936 | OP380513 | *P. coronatus* | GQ482424  GQ482423  AB843693  AB843066  GQ482422 |
| *P. canariensis* | 16,936 | OP380512 | *P. examinandus* | LC087218  LC087217  LC087216  LC087196  LC087186 |
| *P. collybita* | 16,936 | OP380541 | *P. fuscatus* | MG681101  GU572030  GU571556  GQ482432  GQ482431 |
| *P. collybita* | 16,936 | OP380540 | *P. grammiceps* | KU343217 |
| *P. collybita* | 16,936 | OP380539 | *P. griseolus* | GQ482433 |
| *P. collybita* | 16,936 | OP380538 | *P. humei* | GU572031  GQ482436  GQ482435  GQ482434 |
| *P. collybita* | 16,936 | OP380537 | *P. ibericus* | KU870815  KU870814  KU870813  KU870812  KU870811 |
| *P. coronatus* | 16,905 | MK533705 | *P. ijimae* | AB843695  AB843694  AB843078 |
| *P. examinandus* | 16,881 | NC_051526 | *P. inornatus* | GU571559  GU571558  GU571557  GQ482439  GQ482438 |
| *P. examinandus* | 16,884 | MK695927 | *P. maculipennis* | HQ608866 |
| *P. examinandus* | 16,884 | MK695926 | *P. magnirostris* | HQ608867 |
| *P. fuscatus* | 16922 | MG681101 | *P. occisinensis* | HQ608869 |
| *P. ibericus* | 16,936 | NC_071943 | *P. omeiensis* | KU343240  KU343225 |
| *P. ibericus* | 16,936 | OP380532 | *P. orientalis* | JQ175810 |
| *P. ibericus* | 16,936 | OP380531 | *P. poliogenys* | MK598975  JQ176209  JQ176208  KU343246 |
| *P. ibericus* | 16,936 | OP380530 | *P. proregulus* | OR030351  GU572033  GU572032  GQ482446  HQ608861 |
| *P. ibericus* | 16,936 | OP380529 | *P. pulcher* | HQ608864 |
| *P. inornatus* | 16,875 | NC_024726 | *P. reguloides* | OR030352  HQ608865 |
| *P. occisinensis* | 16,879 | MK513447 | *P. schwarzi* | JQ175812  JQ175811  HQ608863 |
| *P. proregulus* | 16,936 | OR030351 | *P. sibilatrix* | GU572035  GU572034  GU571563  GQ482449  GQ482448 |
| *P. proregulus* | 16,880 | NC_037189 | *P. soror* | KU343242  KU343227  KU343226  KU343219 |
| *P. reguloides* | 17,007 | OR030352 | *P. tenellipes* | GQ482453  MG917724  KY627727  LC087226  LC087203 |
| *P. schwarzi* | 16,920 | MK411584 | *P. tephrocephalus* | KU343241  KU343239  KU343238  KU343233  KU343232 |
| *P. sibilatrix* | 16,979 | MN122829 | *P. trivirgatus* | JQ175813 |
| *P. sindianus* | 16,936 | NC_071942 | *P. trochiloides* | OR030353  GU572036  GU571565  HQ608862  GU571564 |
| *P. sindianus* | 16,936 | OP380526 | *P. trochilus* | JF498809  GU572038  KX283148  GQ482468  KU722442 |
| *P. sindianus* | 16,936 | OP380525 | *P. valentini* | MK598984  MK598983  MK598982  MK598981  MK598980 |
| *P. sindianus* | 16,936 | OP380524 | *P. whistleri* | KU343245  KU343243  KU343230  KU343229  KU343221 |
| *P. sindianus* | 16,936 | OP380523 | *P. yunnanensis* | MK598927 |
| *P. tenellipes* | 16,972 | OL628872 | *Aegithalos concinnus concinnus* | KF951091 |
| *P. tenellipes* | 16,904 | NC_045525 |  |  |
| *P. trochiloides* | 16,966 | OR030353 |  |  |
| *P. trochilus* | 16,934 | NC_060851 |  |  |
| *Aegithalos concinnus concinnus* | 17,940 | KF951091 |  |  |
